# Immunotherapy of chimeric antigen receptor NK cells: status and its promising future

**DOI:** 10.3389/fimmu.2025.1608277

**Published:** 2025-10-30

**Authors:** Fang Wang, Zhaoyuan Huang, Xiaoming Feng, Mingxia Shi

**Affiliations:** ^1^ Department of Hematology, The First Affiliated Hospital of Kunming Medical University, Kunming, China; ^2^ State Key Laboratory of Experimental Hematology, National Clinical Research Center for Blood Diseases, Haihe Laboratory of Cell Ecosystem, Institute of Hematology & Blood Diseases Hospital, Chinese Academy of Medical Sciences & Peking Union Medical College, Tianjin, China; ^3^ Tianjin Institutes of Health Science, Tianjin, China; ^4^ T-Cell Precision Therapy Lab, Zhejiang Key Laboratory of Medical Epigenetics, Department of Pathology and Pathophysiology, School of Basic Medical Sciences, Hangzhou Normal University, Hangzhou, China

**Keywords:** chimeric antigen receptors, CAR NK cells, natural killer cells, immunotherapy, NK

## Abstract

Chimeric antigen receptors (CARs) are genetically engineered fusion proteins composed of extracellular antigen-recognition domains and multiple intracellular signaling domains. Although CAR T-cell immunotherapy has achieved significant advancements in treating hematologic malignancies, its application against solid tumors remains less successful. Key challenges—including production complexities, the scarcity of tumor-specific antigens, and limitations in cell trafficking and tumor infiltration—continue to impede therapeutic efficacy. Natural killer (NK) cells, essential innate immune lymphocytes, play a critical role in targeting malignant cells. Their unique antigen-recognition mechanisms, potent cytotoxicity, and favorable clinical safety profile position CAR NK cells as a promising alternative for targeted cancer therapy, especially for solid tumors. However, the transient persistence of NK cells *in vivo* and the technical challenges associated with their preparation currently limit the broader clinical adoption of this approach. This review examines the advantages of CAR NK cells immunotherapy and synthesizes current domestic and international research to advance the understanding of CAR NK cells therapeutics.

## 1 Introduction

Cellular immunotherapy, an innovative approach in oncology often referred to as adoptive cell therapy, has become a major focus in tumor treatment. The expression of chimeric antigen receptors (CARs) on immune cells has revolutionized immunotherapy by enabling the redirection of a patient’s immune cells against malignancies. Global research efforts in CAR T-cell therapies have resulted in more than 1,000 clinical trials to date. Since 2017, the U.S. Food and Drug Administration (FDA) has approved six CAR T-cell products for hematologic malignancies (**Table 1**). Despite these advances, CAR T-cell immunotherapies confront several limitations: intricate manufacturing processes, insufficient tumor-specific antigen targets, and suboptimal cellular trafficking and tumor penetration. Furthermore, post-treatment complications including antigen escape and treatment-related toxicities may worsen disease progression and sustain therapeutic shortcomings.

**Table 1:** FDA approved CAR T product.

Name	Target	Structure	Indications	Approve Time	References
Kymriah	CD19	CD3ζ and 4−1BB	B-ALL	Aug 30,2017	(1)
			R/R DLBCL	May 1,2018	(2)
			R/R FL	May 27,2022	(3)
Yescarta	CD19	CD3ζ and CD28	LBCL	Oct 18,2017	(4)
			FL	Mar 5,2021	(5)
			DLBCL	Apr 1,2022	(6)
Tecartus	CD19	CD3ζ and CD28	MCL	Jul 24,2020	(7)
			B-ALL	Oct 1,2021	(8)
Breyanzi	CD19	CD3ζ and 4−1BB	LBCL	Feb 5,2021	(9)
			CLL/SLL	Mar 21,2024	(10)
			FL	May 15,2024	(11)
			MCL	May 30,2024	(12)
Abecma	BCMA	CD3ζ and 4−1BB	MM	Mar 26,2021	(13)
Carvykti	BCMA	CD3ζ and 4−1BB	MM	Feb 28,2022	(14)

Natural killer (NK) cells, cytotoxic lymphocytes of the innate immune system, represent 5-10% of peripheral blood lymphocytes and originate from lymphoid progenitors. These cells possess a distinctive antigen recognition system that operates independently of the MHC-I restriction. Genetic modification of NK cells to express CARs augments their tumoricidal activity, refines target recognition, and circumvents immune evasion strategies-establishing CAR NK cells therapy as an emerging frontier in cancer immunotherapy. In contrast to CAR T cells, CAR NK cells offer three key advantages: (1) The MHC-unrestricted target recognition, (2) multiple potential cell sources, and (3) reduced treatment-related toxicity, making them applicable to both hematologic and solid tumors. This review systematically evaluates the benefits and limitations of CAR NK cells therapy, synthesizes current research progress, and identifies remaining technical challenges requiring resolution.

## 2 CAR structure

The structural architecture of chimeric antigen receptors (CARs) comprises four essential components: (1) single-chain variable fragments (scFv), (2) hinge regions, (3) transmembrane domains (TM), and (4) intracellular signaling domains. When expressed in NK cells, CARs significantly enhance antitumor activity through improved target recognition accuracy and immune evasion resistance (15). The scFv constitutes the antigen-binding domain, formed by the variable regions of immunoglobulin heavy (VH) and light (VL) chains connected via a flexible peptide linker. This domain determines the binding specificity and affinity of the CAR (16). Serving as a structural spacer, the hinge region optimally positions the CAR on the cell surface to facilitate antigen engagement. Common hinge domains derive from CD8, CD28, or IgG molecules, with their length carefully selected based on target antigen accessibility - longer hinges (e.g. IgG-derived) are preferred for membrane-proximal antigens, while shorter hinges (e.g. CD8-derived) suffice for surface-exposed antigens (17).The transmembrane region primarily anchors the CAR to the immune cell membrane, with CD8α and CD28 being the most frequently utilized. The choice of TM domains has been shown to influence CAR functionality and the degree of cellular activation. Notably, CD28 TM domains preferentially induce activation-induced cell death (AICD) in T cells compare to the CD8α-derived TM domain, while the CD3ζ-derived TM domain facilitate CAR dimerization with endogenous TCR complexes, enhancing T cell activation (18).

The evolution of CAR structure has largely centered on refining intracellular signaling structural domains. The first generation of CARs contained only one activation region, namely CD3ζ. Subsequent generations incorporated additional co-stimulatory regions. Commonly used co-stimulatory molecules include CD28 and 4-1BB.CD28 induces a induce stronger acute response but with shorter lasting time, whereas 4-1BB has a more limited but sustained stimulation capability. Other molecules such as OX40 (19), CD27 (20) and inducible T-cell co-stimulation (ICOS) (21),are also being tested in new research, although their efficacy remains comparable to CD28 and 4-1BB.

## 3 Special structure on NK cells

Initial CAR structures for NK cell engineering were similar to those designed for T-cell therapy and lacked specificity for NK cells because they tailored T cells. To maximize the efficacy of NK cell signaling, it is essential to explore more optimized and specific co-stimulatory domains.

First-generation CAR NK cells, like CAR T cells, utilized only CD3ζ as a single activated intracellular signaling domain (22). Second-generation CARs incorporated an additional co-stimulatory domain, such as CD28 or 4-1BB, while third-generation CARs featured multiple co-stimulatory domains (23). Co-stimulatory molecules are typically derived from the CD28 family (including CD28 and ICOS), the tumor necrosis factor receptor (TNFR) gene family (including 4-1BB, OX40, and CD27), or the signaling lymphocyte activation molecule (SLAM)-related receptor family (including 2B4) (24). Although some signaling/co-stimulatory domains used in CAR design (e.g. CD3ζ and 4-1BB) are shared by NK cells and T cells, it is necessary to identify more specific co-stimulatory regions (e.g., DAP10, DAP12, or 2B4) based on the characteristics of NK cells to maximize the cytotoxic potential of NK cells, as they are regulated by their own activating and inhibitory receptors. DAP10 and DAP12 adaptor molecules containing immunoreceptor tyrosine-based activation motifs (ITAMs) that transmit activation signals to NK cells. DAP10 is essential for signaling through the activation receptor NKG2D, while DAP12 mediates signaling through NKG2C, NKp44, and killer immunoglobulin receptor (KIR) activation (25). NK cells transduced with anti-CD19 CARs containing either the DAP10 or CD3ζ signaling domains have been shown to successfully induce NK cytotoxicity, but optimal responses were achieved when both domains were included in the CAR construct (26). Similarly, CAR NK cells targeting prostate stem cell antigen (PSCA) exhibited enhanced cytotoxicity when DAP12 was doped into the CAR construct compared to results with CD3ζ alone (27). 2B4 is another activating receptor belonging to the SLAM family of proteins. Upon binding to its natural ligand CD48, 2B4 mediates signaling through its ITSM, which recruits bridging molecules such as SLAM-associated proteins (SAPs). 2B4 binds to CD48 on target cells and induces NK cell activation resulting in increased cytotoxicity and IFN-γ production (28). Recently, the fourth-generation CAR NK cells carrying transgenic “payloads” (e.g. IL-2 or IL-15) have been developed to enhance the proliferation, longevity, and activity of CAR NK cells, as well as their cytotoxicity against antigen-negative tumor cells.

## 4 Mechanisms of NK cell cytotoxicity

**Figure 1:**
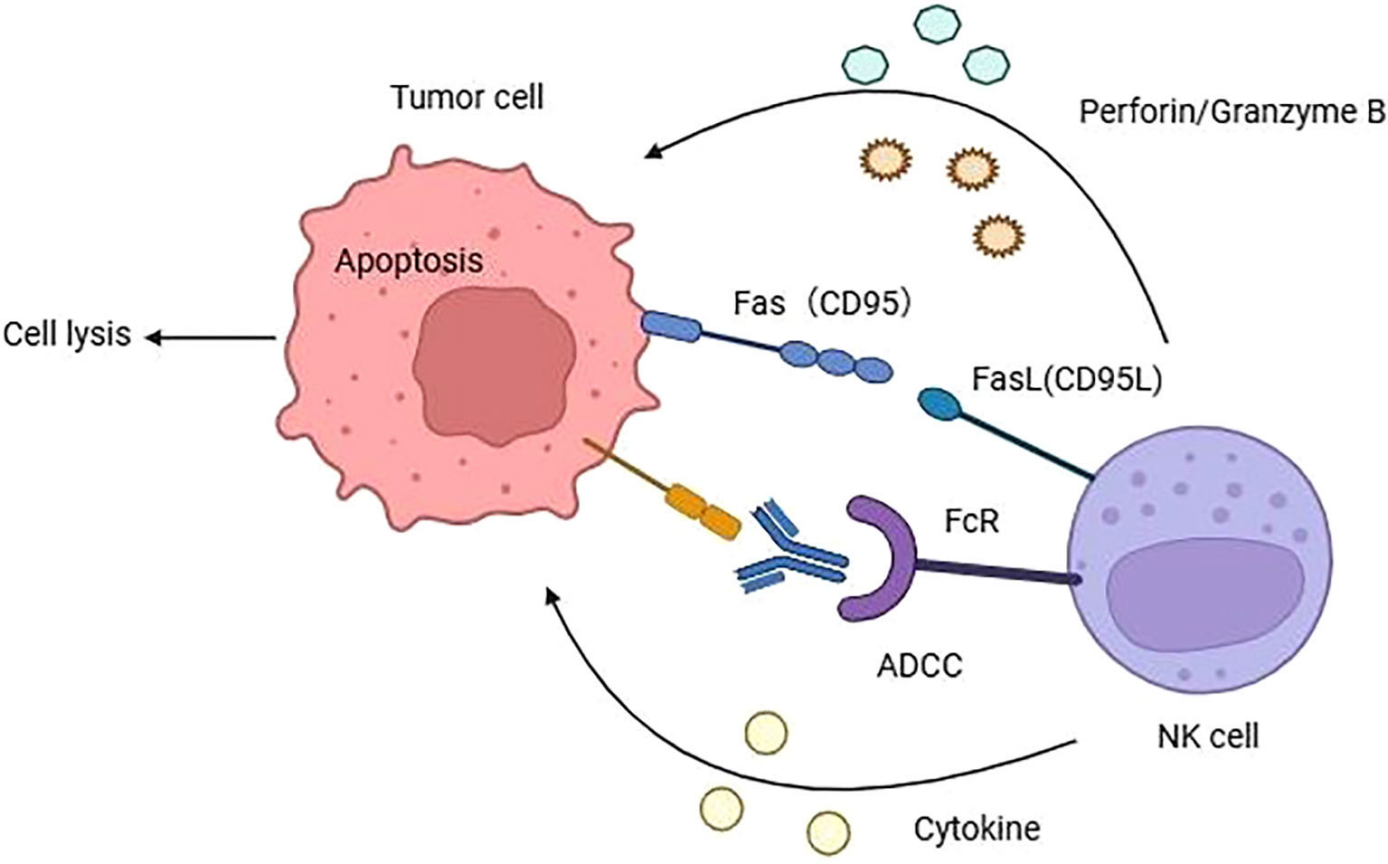
The mechanisms of NK cells mediated cytotoxicity to cancer cell. Natural killer cells primarily exert their cytotoxic effects through four ways: (1) NK cells can secrete perforin and granzyme B to induce cell lysis. (2) NK cells elicit apoptosis through binding to death ligands located on the cell membrane surface. (3) NK cells can recognize the Fc of IgG antibodies on cancer cells via CD16, activating NK cells to secrete toxic substances and trigger cell apoptosis. (4) NK cells can secrete various cytokines such as IFN-γ, TNF-α, IL-2 and so on.

The mechanisms of NK cells mediated cytotoxicity are diverse and can be summarized as follows (**Figure 1**): (1) The perforin/Granzyme B Pathway: NK cells release granzyme B and perforin, which directly form transmembrane channels on the target cell membrane, enhancing membrane permeability and causing target cell lysis. This pathway is crucial for eliminating mutant tumor cells (29). (2) The death ligand pathway: NK cells typically express three distinct death ligands: Fas ligand (FasL), TNF ligand, and TNF-associated apoptosis-inducing ligand (TRAIL), which induce apoptosis by binding to their corresponding death receptors on the surface of tumor cells (30). (3) The cytokine pathway: NK cells secrete various cytokines, including IFN-γ, TNF-α, and IL-2, among others (31). IFN-γ inhibits the proliferation of tumor cells, blocks tumor angiogenesis, and overexpresses MHC molecules in cells, further stimulating antigen presentation. TNF-α activates the nucleic acid-degrading endonuclease in target cells, leading to DNA degradation and the induction of programmed cell death. IL-2, on the other hand, stimulates NK cell proliferation and enhances their killing activity. (4) The antibody-dependent cell-mediated cytotoxicity (ADCC) pathway: CD16, expressed by NK cells, binds to the IgG Fc segment of target cells, inducing activation through signaling and subsequent cell death (32). The administration of IL-2 and IFN-γ markedly augments the ADCC effect of NK cells by inducing Fas expression, which in turn activates the death ligand mechanism and induces apoptosis.

Moreover, NK cells can activate and kill cancer cells by engaging with activating and inhibitory KIRs on the cell surface. In the earliest papers on NK cell activity, the “missing self” hypothesis was proposed, which suggests that the MHC molecules on the surface of healthy cells normally act as ligands for inhibitory receptors, aiding NK cells in establishing self-tolerance and preventing the lysis of healthy cells. Subsequent research identified specific receptors on NK cells that recognize the MHC, which were proven to be KIRs (33). It has been demonstrated that cells under stress and cancer cells down-regulate the expression of MHC I and disengage from the inhibitory KIRs, or up-regulate stress-induced molecules that engage activating KIRs. These molecules participate in KIRs activation, resulting in the activation of NK cells and the elimination of target cells. This can occur either directly through NK cell-mediated cytotoxicity or indirectly through proinflammatory cytokine-mediated killing (34).

## 5 The advantages of CAR NK cells

The success of CAR T-cell therapy in clinical trials has prompted extensive research into CAR NK cells development. Due to their lower risk of side effects and enhanced tumor cytotoxicity, CAR NK cells represent a promising alternative to CAR T-cell therapy. Below we present a comparative analysis of CAR T and CAR NK cells therapies (**Table 2**).

**Table 2:** Comparison of CAR T and CAR NK therapy.

Parameter	CAR T	CAR NK
Cell source	Peripheral blood, iPSC, Autologous T cells or the MHC-matched T cells, Cord blood cells	Peripheral blood cells, NK cell lines, Cord blood cells, iPSC
HLA-matching before allogeneic administration	Required	Not required (possible allogeneic application)
Antitumor mechanism	CAR-dependent Direct cytotoxicity	Direct cytotoxicity CAR-dependent cytotoxicity CAR-independent cytotoxicity ADCC
CAR genome transfection success	Higher success	Lower success
Life span	Longer	Shorter
Safety	Lower	Higher
GVHD	Higher	Lower
CRS	Higher	Lower
ICANS	Higher	Lesser

GVHD, (Graft-versus-host disease); CRS, (Cytokine storm syndrome); ICANS, (Immune effector cell-associated neurotoxicity syndrome).

### 5.1 Wider resources of immune cells

Due to allogeneic reactivity and graft-versus-host disease (GVHD) risks, CAR T cell therapy requires autologous T cells. However, most extensively treated patients exhibit peripheral blood T-cell counts significantly below the required threshold. Consequently, CAR T cell therapy often necessitates longer production timelines, and some patients must temporarily pause treatment to accumulate sufficient cells-a delay that increases risks of disease recurrence and progression. Additionally, hematologic malignancy patients face infection risks when using autologous bone marrow-derived cells. In contrast, NK cells do not require activation by the MHC I pathway and present lower allogeneic reactivity risks, eliminating the strict need for autologous sources. The current sources of NK cells include the NK92 cell line, peripheral blood (PB) cells, umbilical cord blood (UCB), and induced pluripotent stem cells (iPSC).

Numerous research is based on engineering NK-92 cells, which originated from a patient with extranodal NK-cell lymphoma. As a cell line, NK-92 cells exhibit greater expandability compared to other NK cell sources, providing a continuous and reliable supply. However, due to their malignant origin, these cells require irradiation to prevent *in vivo* implantation (35). Studies demonstrate that while irradiated NK-92 cells maintain their cytotoxic activity, their proliferative capacity becomes limited, often necessitating multiple infusions to achieve therapeutic efficacy (36). An additional limitation is the natural absence of the CD16 (Fcγ RIII) domain in NK-92 cells, which renders them incapable of mediating antibody-dependent cellular cytotoxicity (ADCC) - a critical native anti-tumor mechanism of NK cells (23).

The second recourse comes from the peripheral blood (PB) from donors, where mature NK cells can be easily collected without HLA matching, but the portion of NK cells only accounts for 10% of peripheral blood monocyte cells (37). PB-NK cells can be identified as two subgroups: the CD56^bright^CD16^-/dim^ population, which predominantly exhibits an immature phenotype, and the CD56^dim^CD16^+^ NK cells, which exhibit a more mature phenotype.CD56^bright^CD16^-/dim^ PB-NK cells acquire CD16 and KIR phenotypes during progressive maturation, but lose the NKG2D phenotype (38). Autologous KIR/HLA matching between NK cells and tumor cells, in addition to immunosuppressive conditions, results in NK cell inactivation and dysfunction. Consequently, allogeneic transplantation preserves a greater degree of NK cell activity in comparison to allogeneic transplant. The heterogeneous nature of PB-derived CAR NK cells, coupled with the difficulty in standardizing them, presents a significant challenge in their clinical application (39). On the other hand, the cellular responses obtained from PB were more efficient and exhibited a longer circulation time than NK cells from alternative sources (40). Moreover, its source is more straightforward, and the acquisition cost is lower.

Separating NK cells from cord blood (CB) is another possible source of materials. NK cells nearly constitute 30% of cord blood lymphocytes (39). CB NK cells are regarded as exhibiting a more naïve phenotype and function compared to PB NK cells (41). Due to their immature nature, CB NK cells demonstrate a restricted capacity for cytotoxicity but, display a robust proliferative capacity and a high susceptibility to cytokine stimulation. Second, CB NK cells contain a lower proportion of T cells when compared to other graft sources, thereby reducing the risk of graft-versus-host disease (GVHD).Even though CB NK cells can be an alternative production, they require a sufficient volume to undergo massive cell division in order to obtain a sufficient number of cells for infusion (42).

The last kind of NK cells comes from iPSCs, which is a recently discovered source. NK cells derived from iPSCs technology represent an optimal source for the development of off-the-shelf CAR NK cells product, given their capacity for unlimited proliferation (43). The primary benefit of iPSC-derived CAR NK cells is their capacity to produce a substantial number of homogeneous CAR NK cells from a single iPSC. Nevertheless, this methodology results in the generation of cells with an immature, less cytotoxic phenotype, analogous to that observed in UCB-derived NK cells (44). Therefore, additional modification of iPSC-derived NK cells is essential to improve their cytotoxicity and survival *in vivo*. The development of iPSC-derived NK cells is still in its early stages, and numerous challenges remain to be addressed before the production of marketable products can be achieved.

### 5.2 Treatment related toxicity

The second advantage of CAR NK cells therapy is the low risk of treatment-related toxicity. CD4^+^ and CD8^+^ T cells play a crucial role in tumor immune surveillance by recognizing tumor-associated antigens (TAAs), which lead to the secretion of various cytokines and chemokines. This suggests that CAR T cells are more susceptible to treatment-associated toxicities, such as cytokine release syndrome (CRS) and central neurotoxicity, than CAR NK cells (45).

CAR T cells activation results in the release of numerous Inflammatory cytokines, caused cytokine release syndromes (CRS) and neurotoxicity. The second CAR T production approved by FDA, Ciltacabtagene autoleucel, showed its cytotoxicity in different clinical trials, with CRS incidents over 90%(Legend-2 trial 90%, CARTITUDE-1 94.8%, CARTITUDE-2 cohort-A 95%, CARTITUDE-2 cohort-B 84.2%), and the incidence of grade ≥3 CRS was 7%, 4.1%, 10%, and 5.5%, respectively. The incidence of ICAN was 1.8%, 20.6%, 15%, and 5.5%, respectively, and the incidence of grade ≥3 ICAN in the CARTITUDE-1 trial was 10.3% (14, 46). Other CAR T productions show the similar cytotoxicity profiles. In contrast, when using CB derived CAR NK cells in the phrase I/II clinical trial for B-cell lymphoma, only one patient happened grade I CRS among 37 patients, and no neurotoxic symptoms were found in all patients (47). In another clinical trial engineered targeting HER-2 CAR NK cells therapy for glioblastoma, 9 patients received treatment with no dose-limiting toxicities, and no patients developed CRS or immune effector cell-associated neurotoxicity syndrome (ICANs). In a recent clinical trial of 86 patients with B-cell lymphoma, 1 of 18 patients (6%) not treated with rituximab-based combination chemotherapy (highest grade 1) and 9 of 68 patients (13%) treated with rituximab-based combination chemotherapy (6 highest grade 1, 3 grade 2) experienced cytokine release syndrome. No instances of neurotoxicity were observed in either group (9).

### 5.3 Lethal effect

As reviewed earlier, we recognize the numerous mechanisms by which NK cells can recognize target tumor cells. NK cells are a group of cytotoxic lymphocytes belonging to the innate immune system. They are capable of rapidly, spontaneously, and efficiently performing cytotoxic functions against tumor and virus-infected cells without any prior sensitization. In contrast to T cells, which recognize MHC-presented antigens, NK cells can directly recognize target cells without the existence of MHC. During tumorigenesis, immune escape can be attributed to the absence of cell surface MHC I molecules. Of the CAR T-cell therapies for diffuse large B-cell lymphoma (DLBCL), Tisagenlecleucel reported a best-ever remission rate of 52% (complete response [CR] rate of 40%) (2), and axicabtagene ciloleucel reported a best-ever remission rate of 83% (CR rate of 58%) in patients (4).The current CAR NK cells clinical trials have predominantly focused on CD19+ targeting B-cell lymphomas. In a clinical trial of CD19^+^ CAR NK cells for B-cell lymphoma, there was a 41% objective remission rate for diffuse large B-cell lymphoma (DLBCL). A 38% ORR (25% CR rate) was also reported in another CD19^+^ CAR NK cells clinical trial. In that particular trial, 9 out of 20 patients diagnosed with large B lymphoma who had undergone CD19^+^CAR T therapy demonstrated a positive response (45%), with a CR rate of 30%. Another clinical trial of CD19^+^ CAR NK cells targeting lymphoma, reported an ORR of 73% (CR rate 64%). It is noteworthy that CAR NK cells demonstrated a response in both low-grade NHL cases and maintained a one-year CR rate of 83% (48). Presently, the available clinical trial data on CAR NK cells therapy is limited in scope and sample size. The validity of the remission rate data comparisons remains to be substantiated. However, given the observed efficacy of CAR NK cells in patients who have undergone CAR T therapy, CAR NK cells emerge as a potential alternative treatment option in cases where CAR T therapy has failed.

### 5.4 Limited lifespan

The average lifespan of NK cells is about 2 weeks (49). In the event of off-target toxicity, the CAR may serve to limit the extent of the adverse reaction, given that its efficacy diminishes over time. However, this also presents a potential challenge, as it may necessitate repeated infusions of CAR NK cells to prolong remission. Such an action could have other potential adverse effects.

## 6 Disadvantages and improvements of CAR NK cells

### 6.1 CAR NK cells preparation

Currently, two principal vectors are employed in the context of CAR gene transduction: viral-based systems and non-viral systems. Viral vectors primarily consist of lentiviruses and retroviruses. Historically, the transduction efficiency of primary NK cells using both retroviruses and lentiviruses has been relatively low. Retroviral vectors are capable of carrying transgenes of an appropriate size (7–8 kb) and have been observed to exhibit high transduction efficiencies (43-93%) in primary PB NK cells (23). However, retroviral vectors cannot steadily express in cells, and retroviruses can only infect cells in the division phase. In a recent study, Muller et al. observed that the CAR NK cells transduction efficiency of RD114 α-retrovirus was approximately three times higher than that of VSV-G lentivirus in primary NK cells on day 3. While both retroviruses and lentiviruses exhibited similar performance from day 7 onwards (50),CAR expression is sustained for at least 2 weeks. Although stable CAR expression can be achieved in NK cells for extended periods using various retroviruses, concerns regarding the safety of retroviral systems persist, particularly when compared to the safer lentiviral systems.

Lentiviral transduction is another commonly employed approach. Lentivirus-based vectors can infect both circulating and non-circulating cells carrying larger transgenes, but their efficiency is often hindered by the natural resistance of NK cells to viral infection. To improve the transduction efficiency of large numbers of antiviral NK cells, researchers have utilized chemicals such as polystyrene (51) and vectofusin-1 (52). A recent study suggested that inhibition of intracellular antiviral defense mechanisms could improve the efficiency of lentiviral transduction in human NK cells (53). Clark et al. found that the 3-phosphoinositide-dependent kinase 1 (PDK1) inhibitor BX-795 negatively controlled the RIG-I, MDA-5, and TLR3 pathways involved in the antiviral response, resulting in an average 3.8-fold increase in lentiviral vector transduction efficiency (54). In addition, rosuvastatin was found to increase VSV-G lentivirus transduction in NK cells by upregulating LDLR levels (55). Altering the pseudotype of the virus (e.g. for BaEV) is also thought to improve the efficiency of viral transduction. Colamartino et al. demonstrated an effective and robust method for NK cell transduction using baboon-enveloped pseudotyped lentivirus (BaEV - lv) (56). They achieved a transduction rate of 23.0 ± 6.6% (mean ± SD) in freshly isolated human NK cells (FI-NK) and 83.4 ± 10.1% (mean ± SD) in NK cells obtained by the NK cell activation and expansion system (NKAES), maintained transgene expression for at least 21 days. Transduction with BaEV-LVs encoding CAR-CD22 resulted in robust CAR expression on 38.3% ± 23.8% (mean ± SD) of NKAES cells and showed specific killing of NK-resistant pre-B-ALL-RS4. In addition, Bari et al. noticed that modified BaEV-LVs exhibited 20-fold or higher transduction rates than VSVG pseudo-type lentiviral vectors (57).

In comparison to viral-based approaches for gene modification, non-viral gene modifications are generally more stable, easier to synthesize, and capable of transduce larger genetic loads (> 100 kb). Currently, non-viral vectors are recognized as a more effective alternative technologies than viral-based in CAR integration. While the electroporation efficiency of DNA is low, it can reach 80-90% transduction rate in primary NK cells by prior activating NK cells with mRNA-based plasmids (58). However, a significant drawback of using electroporation to achieve CAR expression is the short duration of expression in the cells. CAR constructs expressed on cell surface of primary NK cells typically sustain for only 3–5 days before losing expression, which greatly shorten the therapeutic window (59).

Another non-viral transduction method involves CRISPR-associated transposon. Transposon-based systems allow for introduction of CAR transgenes at predetermined sites with higher efficiency, representing a significant advantage over traditional methods that lack integrating elements. However, despite their improved transduction efficiency and increased gene stability, transposon interventions still require viral or electroporation methods for gene modification. We anticipate that with further development of transposons and transfection methods, the use of transposons to generate CAR NK cells will become a more feasible gene-editing tool.

### 6.2 Short lifespan

NK cell growth is cytokine-dependent, and previous clinical trials have confirmed that infused NK cells often become undetectable within 1–2 weeks post infusion (60). In the absence of cytokine support, the shortened *in vivo* lifespan of NK cells reduces treatment-related toxicities but also narrows the therapeutic window. What’s more, the low *in vivo* persistence may lead to early disease relapse. Consequently, multiple rounds of infusion are often required to achieve the desired effect, leading to potential adverse effects. A promising solution to this challenge is the modification of NK cells with transgenic genes encoding cytokines expressed at the membrane or constitutively released. Several studies have shown that NK cells conjugated with the IL-2/IL-15 transgene or CAR constructs supplemented with IL-15 improve proliferation and persistence without causing cytotoxicity (25). Additionally, it has been demonstrated that cytokine-induced memory-like (CIML) NK cells differentiate following transient pre-activation with interleukin-12 (IL-12), IL-15, and IL-18, and exhibit enhanced responses to restimulation with cytokines or activated receptors weeks to months after pre-activation. In the phase I clinical trial evaluating CIML NK cells for relapsed-refractory AML, CIML NK cells were remained detectable *in vivo* one week after the infusion, with a large predominance, and showed a strong killing effect on leukemia cells (60).

### 6.3 Transportation and tumor infiltration

One of the principal challenges of CAR immunotherapy is its limited capacity for CAR NK cells to effectively deliver and home to basal cells, a factor intrinsically linked to the efficacy of transferred NK cells. This aspect is particularly crucial in the context of solid tumor therapy. The rapid homing of NK cells to the tumor bed is controlled by complex interactions between NK cells and chemokines released by tumor cells. The major chemokine receptors expressed in the NK cell population, including CXCR4, CXCR3, CCR3, CCR5, and CX3CR1, facilitate the distribution of NK cells in response to chemokines present in the tumor microenvironment (TME) (61).

Müller et al. demonstrated in a glioblastoma mouse model that anti-EGFRvIII CAR NK cells engineered to produce CXCR4 displayed selective chemotaxis towards CXCL12/SDF1-secreting glioblastoma cells, resulting in enhanced tumor regression and survival (62).Besides, the modification of NKG2D CAR NK cells to express CXCR1 significantly increased anti-tumor responses in murine peritoneal ovarian cancer xenografts (63). Schomer et al. demonstrated that CCR7-engineered CD19 t-haNK cells could effectively execute targeted killing and ADCC effects on drug-resistant lymphoma cells. Moreover, when CCR7 CD19 t-haNK cells injected into NSG mice inoculated with CCL19-secreting Raji lymphoma cells, tumor control and survival were increased in both local and systemic tumor models compared to mice treated with CD19 t-haNK cells that do not express the CCR7 receptor and control mice. Interestingly, larger subcutaneous tumors (>150 mm^3^ at the start of treatment) appeared to be more sensitive to CCR7 CD19 t-haNK injections than smaller ones (64).

Except for cytokines, NK cells can also enhance targeted killing of tumor cells through interacting with adhesion molecules such as selectins. Hong et al. treated cells with human fucosyltransferase 6 (FUT6) (33, 43–45) and GDP- fucose, generating the cell surface E-selectin ligand sialyyl Lewis X (sLeX) to promote migration into the bone marrow (BM). Experimental results showed that bone marrow colonization of (BPC Neu5Ac)-sLe X -NK-92MI cells was increased by approximately 60% compared to BPC-less FUT6-treated Neu5Ac-NK-92MI cells (65).

These findings underscore the importance of optimizing the immunochemical properties of the CAR construct and enhancing the trafficking of transferred cells to the tumor, which are critical for improving the efficacy of CAR NK cells therapies.

### 6.4 Tumor immune microenvironment

TME is a complex ecosystem orchestrated by the cancer cells and comprised of various cellular components from both the tumor and the host, including immune cells, fibroblasts, endothelial cells, inflammatory cells, and lymphocytes), extracellular matrix (ECM), vasculature, and chemokines. The dynamic interactions between cancer cells and these components of the TME is crucial for tumor cells to generate heterogeneity, clonal evolution and enhanced multi-drug resistance (66). Hypoxia is a critical factor within the TME, with cancer cell hypoxia-inducible factor-1 (HIF-1) initiating the activation of several factors to commence the angiogenic process (67), This includes the principal angiogenic ligand, vascular endothelial growth factor (VEGF), and its corresponding receptors, such as VEGFR2, to adapt to hypoxic conditions. HIF-1 has also been found to activate the transforming growth factor β (TGF-β), WNT, and NOTCH signaling pathways while inhibiting the Hippo signaling pathway, thereby promoting the survival of tumor stem cells (68–70). Shaim et al. have shown that treating allogeneic NK cells with integrins or TGF-β signaling inhibitors, or engineering TGFBR2 gene-edited allogeneic NK cells, can prevent GSC-induced NK cell dysfunction and tumor growth in mice transplanted with GSCs. These findings have unveiled important mechanisms by which GSCs evade NK cell immunity, suggesting that the αv integrin/TGF-β axis could be a potentially useful therapeutic target in glioblastoma multiforme (GBM) (71). Furthermore, Klopotowska et al. have discovered that peroxiredoxin-1 (PRDX1) is a deficient element in the antioxidant defense of NK cells. They constructed PD-L1-CAR NK cells overexpressing PRDX1 and applied them in a mouse breast cancer cell model, observing a potent anti-tumor activity of these engineered CAR NK cells (72).

Another key contributor to TME-induced NK cell exhaustion is the interaction with molecular checkpoint proteins (73). Zhang et al. noticed that TIGIT was associated with NK cell depletion in tumor-bearing mice and colon cancer patients. They found that treatment with a monoclonal antibody against TIGIT in models of colon cancer, breast cancer, and melanoma resulted in increased NK cell infiltration and significant inhibition of tumor growth. TIGIT deficiency was shown to prevent NK cell depletion and enhance host anti-tumor immunity (74).Daher et al. constructed CD19 CAR NK cells targeting the cytokine-containing inducible Src homolog 2 (CIS) protein. Results indicated that CB NK cells transduced with a fourth-generation vector encoding anti-CD19 CAR and interleukin 15 (IL-15) induced greater *in vivo* expansion and longer persistence than un-transduced (NT) NK cells (75).

These findings highlight the potential for engineering NK cells to overcome TME-induced challenges and enhance the efficacy of immunotherapies.

### 6.5 Side effects

Similar with other therapeutic modalities, CAR NK cells therapy also is associated with adverse effects. However, as previously stated, the limited lifespan of NK cells implies a reduced likelihood of their infiltration into normal tissues such as the lungs and liver. This reduces the probability of GvHD and CRS associated with CAR NK cells therapy. The most prevalent adverse effects of CAR NK cells therapy include fever and fatigue, which are attributable to elevated serum levels of C-reactive protein (CRP) and IL-6. In a recently clinical trial by David et al. investigating CD19+ CAR NK cells therapy for B-cell tumors, no instances of neurotoxicity or graft-versus-host disease were observed among the 33 patients, and only one patient developed CRS (grade I). Lymphocyte-depleting chemotherapy resulted in reversible hematologic toxicity across all patients, without reaching the maximum tolerated dose (47).

Despite the low incidence of adverse effects, there is scope for further enhancing the safety profile of CAR NK cells therapy. One such strategy involves the incorporation of suicide genes, which can be induced in response to adverse events. This approach has been realized using the HSV-TK/GCV suicide system and the CRISPR/Cas9 apoptosis pathway, as demonstrated in previous studies (76). In a preclinical report by Liu et al, CAR NK cells were transfected with a construct comprising CD19-CAR, inducible caspase-9 (iC9), and a transgene encoding IL-15. Post administration, the activation of iC9 resulted in the successful elimination of CAR NK cells, providing a safety switch for managing adverse reactions (77).

These findings highlight the ongoing efforts to minimize the side effects of CAR NK cells therapy, thereby improving its safety and efficacy in clinical settings. The integration of safety switches in CAR NK cells therapy represents a promising avenue for the precise control of treatment-related adverse effects.

## 7 Current status of CAR NK cells therapies

Clinicaltrials.gov currently has published 140 clinical trials of CAR NK cells therapy. Most of these CAR NK cells therapies still concentrate on hematologic tumors, but clinical trials with new targeting sites for solid tumor are under investigation (**Supplementary Table 1**).

### 7.1 Preclinical application of CAR NK cells in hematologic tumors

The notable success of CAR T therapy in hematology has prompted a substantial focus on CAR NK cells therapy, albeit with limited success. In addition to the successful targets that have been well-established in the context of CAR T therapy, researchers are continually exploring new targets with the aim of improving the treatment of hematologic diseases.

NKG2D is a critical activating cell surface receptor that identifies and eradicates infected and cancerous cells by engaging a range of stress-inducing ligands, including NKG2DL, MICB, and ULBP. NKG2D ligand expression has been observed in over 70% of human cancers. Alejandra et al. engineered CAR NK cells that target NKG2D for the treatment of multiple myeloma (MM), compare to the group of memory-like CAR T-cells transduced with NKG2D, the mice that received the CAR NK cells demonstrated prolonged disease-free survival and exhibited a significant reduction in plasma cells in the bone marrow (78).

CD70, a member of the tumor necrosis factor superfamily, is a type II transmembrane protein, variety of tumors reported the expression of CD70. Guo et al. treated B-cell lymphoma by constructing UCB-derived CAR NK cells carrying IL15 cytokine targeting CD70, and found that carrying IL15 could promote the survival of CAR NK cells *in vivo*. Furthermore, the infusion of CD70 CAR NK cells into mice inoculated with lymphoma cells demonstrated that CAR NK cells targeting CD70 exhibited not only effectiveness against CD19^+^ tumor cells but also a certain degree of efficacy against CD19^-^ tumor cells, thereby demonstrating superior efficacy in comparison to CAR NK cells targeting CD19. Furthermore, following two infusion attempts with CAR NK cells, the mice demonstrated a tumor-free growth period of over 100 days. At the 113th day after the initial infusion, the presence of CAR NK cells was still detected in the peripheral blood, liver, spleen, bone marrow, and other organs during a necropsy. The long-term survival of CAR NK cells *in vivo* was demonstrated (79).

In recent years, memory-like NK cells have been found to exhibit enhanced proliferative properties and rapid reactivation with augmented toxicity upon re-exposure to immunogens. Han Dong et al. constructed a chimeric antigen receptor (CAR) memory-like NK cell targeting the NMP1 CAR.ASCT-2 is highly expressed in CIML NK cells; therefore, baboon endogenous retroviral envelope BaEV was used to achieve efficient assembly of CAR NK cells.NPM1c-CAR-CIML NK cells demonstrated high specificity and potent anti-leukemic activity *in vitro* and in preclinical human xenograft models (80).

### 7.2 Clinical application of CAR NK cells in hematologic tumors

At the time of writing, there are 140 registered clinical trials, with 70% of these studies focusing on hematologic tumors. The use of CAR NK cells therapy is still in the experimental phase. At the time of writing, only 4 clinical trials are searchable on PubMed.

The first trial is a phase I clinical study investigating the efficacy of CD33-CD28-4-1BB CAR NK-92 cells in the treatment of acute myeloid leukemia (AML). Two patients received three doses of CAR NK-92 cells infusions of 3×10^8^, 6×10^8^, and 10^9^, and one patient received infusions of 10^9^, 3×10^9^, and 5×10^9^.All three patients exhibited a notable elevation in temperature on the second day following CAR NK cells infusion. Two patients demonstrated grade I CRS, while one patient did not respond to treatment. No discernible neurotoxicity was observed in any of the three patients. The results demonstrated that infusion doses of CD33-CAR NK-92 cells up to 5 × 10^9^ cells per patient could be safely applied without obvious adverse effects (81).

The second study examined the use of cord blood-derived HLA-mismatched CD19 CAR NK cells to treat patients with CD19^+^ NHL or CLL. A total of 11 patients were treated with HLA-mismatched anti-CD19 CAR NK cells derived from umbilical cord blood. Of these patients, 8 (73%) achieved remission, with 7 (4 lymphomas and 3 CLL) exhibiting complete remission and 1 (Richter’s transformation component remission) exhibiting persistent CLL. The response was rapid, observed within 30 days after infusion of all dose levels. The infused CAR NK cells demonstrated evidence of expansion and persistence at low levels for a minimum of 12 months. The majority of the 11 patients with relapsed or refractory CD19-positive cancers responded positively to CAR NK cells therapy, with minimal adverse effects (82).

The third study is a clinical trial of umbilical cord blood-derived CD19 CAR NK cells for the treatment of B-cell tumors. The objective is to ascertain the safety and efficacy of the therapy. A total of 37 patients with relapsed or refractory CD19^+^ B-cell malignancies were enrolled in the trial. Regarding safety, no patients exhibited neurotoxicity or GVHD, and only one developed CRS (grade I). All patients exhibited hematologic toxicity, which was reversible with lymphodepleting chemotherapy. The maximum tolerated dose was not reached. In terms of efficacy, the OR rate, including partial remission (PR) and CR, was 48.6% (18/37) at day 30 and day 100 in the 37 patients enrolled in the study. The CR rate was 27% (10/37) and 29.7% (11/37) at day 30 and day 100 in the 37 patients. The one-year CR rate was 37.8% (14/37). The median time to the initial response was 30 days, with a range of 30 to 55 days. Of the 10 patients who achieved CR at day 30, nine remained in CR at day 180. Additionally, four of the eight patients who achieved PR at day 30 ultimately achieved CR. The probability that a patient who achieved CR at day 30 post-infusion would remain in CR at 12 months was 70.0% (46).

### 7.3 Preclinical studies of CAR NK cells in solid tumors

A search for clinical trials using the keyword “CAR NK” returned 167 results. Of these, 25 were for trials involving solid tumors. However, only a fraction of these trials had results reported in PubMed. A multitude of preclinical applications exist for various systemic solid tumors, as evidenced by the studies reviewed below.

Li et al. developed a CAR NK cells model against claudin-6(CLDN6) target for ovarian cancer treatment. Anti-CLDN6 CAR NK cells demonstrated a more pronounced anti-tumor effect compared to CD19 CAR NK cells. Furthermore, the engineered anti-CLDN6 CAR1, comprising NKG2D and 2B4 within the CAR configuration, exhibited enhanced cytokine secretion and degranulation when compared to the anti-CLDN6 CAR2, which incorporated CD28 and 4-1BB within the CAR structure. Mesothelin, a differentiation antigen present on the surface of normal mesothelial cells and a variety of tumor cells, is recognized by monoclonal antibodies. Another study targeting ovarian cancer was conducted by developing mesothelin-targeted, human embryonic stem cell-derived CAR NK cells. The researchers used a cryopreservation method tailored for CAR-iNK cells that allowed the CAR NK cells to maintain a high level of tumor-killing effect even after six months of cryopreservation (83). CAR NK-92 cells therapies targeting MLN have also been utilized in mouse models of colorectal and gastric cancer, with evidence indicating tumor cell killing effects (84). However, as previously mentioned, NK92 cells require irradiation due to their malignant origin, and these CAR NK cells have been shown to survive in mice for more than 48 hours. However, a significant decline is observed on day 7, necessitating repeated infusions to maintain a prolonged tumor-suppressive effect.

Due to excellent live-cell imaging properties of zebrafish embryos and larvae’s, Nivedha et al. explored the role of CAR NK-92 on metastatic breast cancer through a zebrafish larval xenograft model. The injection of *in vitro* co-cultured PD-L1 CAR NK cells and breast cancer cells into zebrafish larvae resulted in the attenuation of the migration of the breast cancer cells. Furthermore, CAR NK cells that targeted both PD-L1 and ErbB2 exhibited nearly 100% lethality rate in zebrafish larvae, in contrast to the 50% lethality rate observed with non-CAR-transduced NK92 cells. However, zebrafish larvae exhibit an absence of adaptive immunity. Consequently, the survival time of CAR NK cells in the *in vivo* model cannot be accurately determined in the absence of endogenous immunity (85).

CD44V6 is an isoform of the CD44 glycoprotein that is expressed in a variety of malignant tumors, including those of the breast, stomach, and colorectum, as well as head and neck squamous cell carcinoma (HNSCC). Recently, Martin et al. constructed CD44v6 chimeric antigen receptor (CAR) natural killer (NK) cells to target triple-negative breast cancers (TNBC) and explored their activity against a 3D structural breast cancer tumor model. They constructed CAR structures with IL15 to promote CAR NK cells implantation and proliferation, and also included a suicide gene in the CAR structures to allow for the removal of CAR NK cells if needed for treatment, ensuring the safety of CAR NK cells therapy (86). Ioana et al. The CAR structures were transduced using a gamma retroviral vector (gRV) to compare the efficiencies of viral envelope protein-mediated CAR transduction in the endogenous viral envelope of baboons (BaEV), the feline leukemia virus (FeLV, termed RD 114-TR), and the great ape leukemia virus (GaLV). For primary natural killer (NK) cells, BaEV-gRV transduction efficiency reached up to 39.3% at an infectious multiplicity of infection (MOI) of 5. *In vitro* experiments were conducted by the investigators to demonstrate 2 to 3-fold enhancement of the cytotoxic effect of CAR NK cells targeting CD 44 v6 against HNSCC compared to non-engineered NK cells (87).

### 7.4 Clinical trials of CAR NK cells therapy on solid tumors

Although CAR NK cells therapy for solid tumors is still in its infancy, a limited number of articles have begun to describe its clinical application.

In a recent study, Michael et al. conducted a phase I clinical trial of HER-2 CAR NK cells injections in patients with glioblastoma. Nine individuals were enrolled in the study and received single-dose injections of 1 × 10^7^, 3 × 10^7^, or 1 × 10^8^ irradiated CAR NK cells into the surgical cavity margin. Throughout the study, no dose-limiting toxic events, CRS, or ICANS were observed. Five patients showed stable disease following recurrent surgery and CAR NK cells injection over a period of 7 to 37 weeks, while four patients experienced disease progression. Two patients displayed evidence of pseudo-progression at the injection site suggesting a treatment-induced immune response. The median progression-free survival (PSF) for all patients was seven weeks, and the median overall survival (OS) was 31 weeks. This trial demonstrated the feasibility and safety of local intracerebral injection of up to 1 × 10^8^ HER2-CAR NK cells. The NK-92/5.28 cell line used was devoid of neurotoxicity and systemic side effects, thereby laying a theoretical foundation for the application of CAR NK cells therapy in the treatment of glioblastoma (48).

## 8 Conclusion

Currently, CAR NK cells therapy has attracted considerable interest from a range of stakeholders due to its favorable safety profile and potent cytotoxic effects. However, it is crucial to recognize the limitations inherent in this therapeutic approach. Sufficient clinical trial data are still lacking to fully elucidate the broad applicability and long-term response of CAR NK cells in monotherapy or combination therapy. Furthermore, additional challenges must be addressed, including the difficulty in fabrication, the short survival time *in vivo*, the poor homing ability, and the inhibition of the TME.

A substantial body of preclinical evidence has identified potential obstacles to CAR NK cells therapies that could impede their long-term efficacy, leading to tumor resistance. This paper also delineates the various methodologies that are currently being explored with the aim of gaining insight into these mechanisms and the targets involved, in order to enhancing the efficacy, *in vivo* expansion and persistence of NK cells. As previously outlined, CRISPR/Cas9 is an optional gene editing procedure for allogeneic NK cells. This technology has been employed with some success in the preparation of CAR NK cells, enhancing efficacy and alleviating TEM inhibition. It is superior to those of the adverse effects of other combinatorial approaches. In light of these findings, it can be posited that the use of multiple gene editing techniques may prove an efficacious method for the preparation of more effective CAR NK cells samples, thus offering an additional avenue for cancer patients with limited access to treatment.
